# Supplementary material for: Brain Fog and Quality of Life at Work in Non-Hospitalized Patients after COVID-19
Source: Int J Environ Res Public Health. 2022 Oct 6;19(19):12816. doi: 10.3390/ijerph191912816 (PMC9564568; doi:10.3390/ijerph191912816)

Supplemental methods – Statistics.

Quantitative data were demonstrated as medians and interquartile ranges (IQR), as the Shapiro-Wilk test showed a nonnormal distribution. A Kruskal-Wallis test and a post hoc test Dunn test were used to compare nonnormally distributed variables (e.g., number of brain fog symptoms) in three or more groups (QoL at work, Supplementary Figure 1). Friedman's ANOVA was performed for nonparametric repeated measures (e.g., QoL at work in four time intervals), followed by Cochran's Q test (Table 1).

Qualitative data described by numbers and percentages were compared using the χ^2 test (Table 2). Mosaic graphs were used to demonstrate a relationship between the change in QoL at work (a decrease or no change or increase in symptoms 4, 4-12, and >12 weeks after COVID-19 and a baseline QoL, Figure 1). For each time interval, an association between QoL at work and elements of brain fog with a Pearson correlation was evaluated to express this time-dependent relationship (Figure 2).

To reduce the risk of multicollinearity, all brain fog elements that showed an association with deterioration of the QoL at work (at least 1 level on the Likert scale) compared to the value before COVID-19 in the univariate model with $p<0.05$ and did not show a substantial correlation with other independent variables ($r>0.7$), were included in a model [34]. This resulted in the creation of Model A (including problems with performing several independent tasks) and Model B (with problems with recalling new information, Table 4). Furthermore, multivariable logistic regression with a stepwise backward elimination procedure was performed to identify predictors of QoL deterioration 4, 4-12, and >12 weeks after COVID-19. The Akaike information criterion was used to assess the goodness of fit of the models. The Hosmer-Lemeshow test tested model calibration. The association of brain fog elements with deterioration in QoL was expressed as odds ratio (OR) and 95% confidence interval (CI). For each model, the area under the receiver operation characteristic curve (AUC) and the 95% CI were used to evaluate predictive accuracy. Values of $P<0.05$ were considered statistically significant. STATISTICA version 13.0 (Statsoft Inc., Tulsa, OK) was used for the analysis.

Supplementary Table S1. Open-ended questions asked to twelve neurologists who underwent COVID-19 during the first step of creating the questionnaire for patients.

1. How do you understand the concept of brain fog?
2. Did you suffer from brain fog before or after COVID-19?
3. When did the symptoms of brain fog appear after COVID-19 infection?
4. How long did they last?
5. Did you use:
   - the help of specialists (psychiatrist, neurologist, psychologist)?
   - pharmacological treatment?
6. Did you experience problems with concentration? Describe what they were like.
7. Did you notice any mental retardation? Describe in a few words what exactly it was and how long it lasted.
8. Did you notice difficulty remembering information or information from the past?
9. Did you have sleep problems, such as excessive sleepiness, insomnia or nightmares?
10. Did you notice any speech problems (e.g., forgetting words, difficulty understanding speech)?
11. Did you notice a decrease in motivation to act?
12. Did you experience anxiety?

Supplementary Table S2. Exploratory factor analysis of the items of the BF-COVID (questions 1-8) and the Quality of Life at Work Questionnaire (question 9).

Factor	Question	Rotated Factor Loadings	Eigenvalues	Percentage of variance
Multitasking and Quality of life at work	4	0.789	3.001	33.45
	9	0.579		
Communication and orientation	1	0.543	1.076	11.96
	2	0.753		
	3	0.462		
	8	0.715		
Memory	5	0.600	1.056	1.06
	6	0.742		

Supplementary Figure S1. Association between the number of brain fog symptoms and quality of life at work (QoL) before COVID-19 (panel A), in acute (<4 weeks, panel B), subacute (4-12 weeks, panel C) and chronic (>12 weeks, panel D) phases of COVID-19. QoL was evaluated with a 4-point Likert scale, where 0 denoted no symptoms, 1 – mild, 2 – moderate, and 3 – severe symptoms. Values are presented as median (interquartile range), and black points indicate outliers. Kruskal-Wallis test p-value <0.05 for all panels. The post-hoc Dunn test p-values <0.05 are presented in the figures.

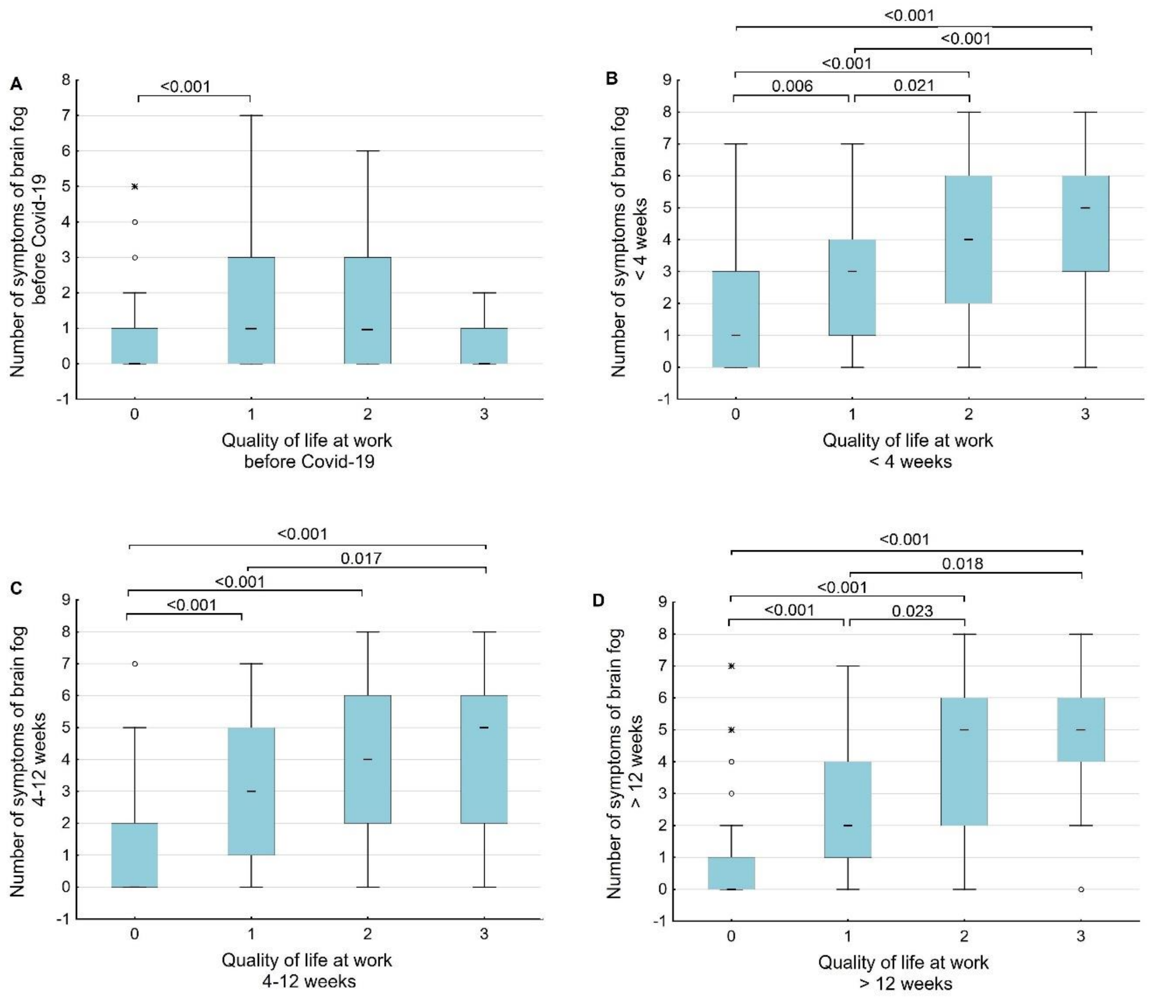

Supplement: Supplementary file 1 [file ijerph-19-12816-s001.zip › ijerph-1883314-supplementary.pdf]
